# Isolated metastasis of hepatocellular carcinoma in the right ventricle

**DOI:** 10.1186/s12872-019-01290-6

**Published:** 2019-12-12

**Authors:** Xin-tong Zhang, Ying Li, Si-hua Ren, Wei-dong Ren, Guang Song, Yang-jie Xiao, Fei-fei Sun, Lu Sun, Xiang-hong Yang, Xue-ying Tan

**Affiliations:** 1 Department of Ultrasound, Shengjing Hospital of China Medical University, sanhao street 36#, Liaoning, Shenyang, 110004 China; 2 Department of Radiology, The First Hospital of China Medical University, najing north street 155#, Shenyang, 110000 China

**Keywords:** Hepatocellular carcinoma, Metastasis, Echocardiography, Right ventricle, Treatment, Inflow obstruction

## Abstract

**Background:**

Hepatocellular carcinoma (HCC) with right ventricle metastasis without inferior vena cava and right atrium involvement is very rare and the prognosis of HCC with RV metastasis is generally poor. The mass in the cardiac chamber may lead to lethal instability of hemodynamics, however, the initial symptom is probably non-specific, which means that diagnosis timely becomes even harder.

**Case presentation:**

We present a 63-year-old male with isolated metastasis of HCC in the right ventricle which caused inflow obstruction. Moreover, we reviewed a series of studies of isolated metastasis of hepatocellular carcinoma between 1980 and 2018, and summarized the relative outcomes.

**Conclusions:**

Isolated metastasis of hepatocellular carcinoma in the right ventricle is extraordinarily rare. It may damage cardiac structure and broke hemodynamic balance. Multimodality imaging plays an important in accurate pre-operation assessment. Nowadays, palliative treatments could relieve fatal symptoms to some degree, however, standard treatment has not been well established.

## Background

Right heart cavity occlusion secondary to metastatic hepatocellular carcinoma (HCC) with cephalad extension via the inferior vena cava (IVC) is rarely reported. The incidence is less than 6% in an autopsy series according to literature review [1]. Furthermore, isolated intracavitary metastasis HCC to the right ventricle (RV) without right atrium (RA) and IVC represents rarer events. The prognosis is poor for metastatic HCC, however, surgical treatment may relief their symptoms and improve life quality. Herein, we report a case of isolated metastasis of HCC in RV diagnosed by multimodality cardiac imaging. Meanwhile we review and summarize similar cases from 1980 to 2019 in English literature and related outcomes.

## Case presentation

A 63-year-old male with a reported history of primary HCC treated with surgery and interventional chemotherapy 2 years ago was admitted to our hospital. He suffered from shortness of breath, abdominal distention and palpitation for 1 month. On physical examination, a grade 3/6 systolic murmur over the pulmonary area was heard. Edema in both lower limbs was observed. ECG demonstrated sinus rhythm, ventricular premature contraction and complete right bundle branch block. Laboratory findings showed that his NT-proBNP level was 1769 pg/ml, CKMB was 27 U/L and TnI was 0.249μg/ml. He had no history of hepatic virus infection. Other findings were albumin33.7 g/L, GGT97U/L, BilT26.4umol/L, BilD10.7umol/L, uric444 umol/L and CRP114mg/L.

**Fig. 1:**
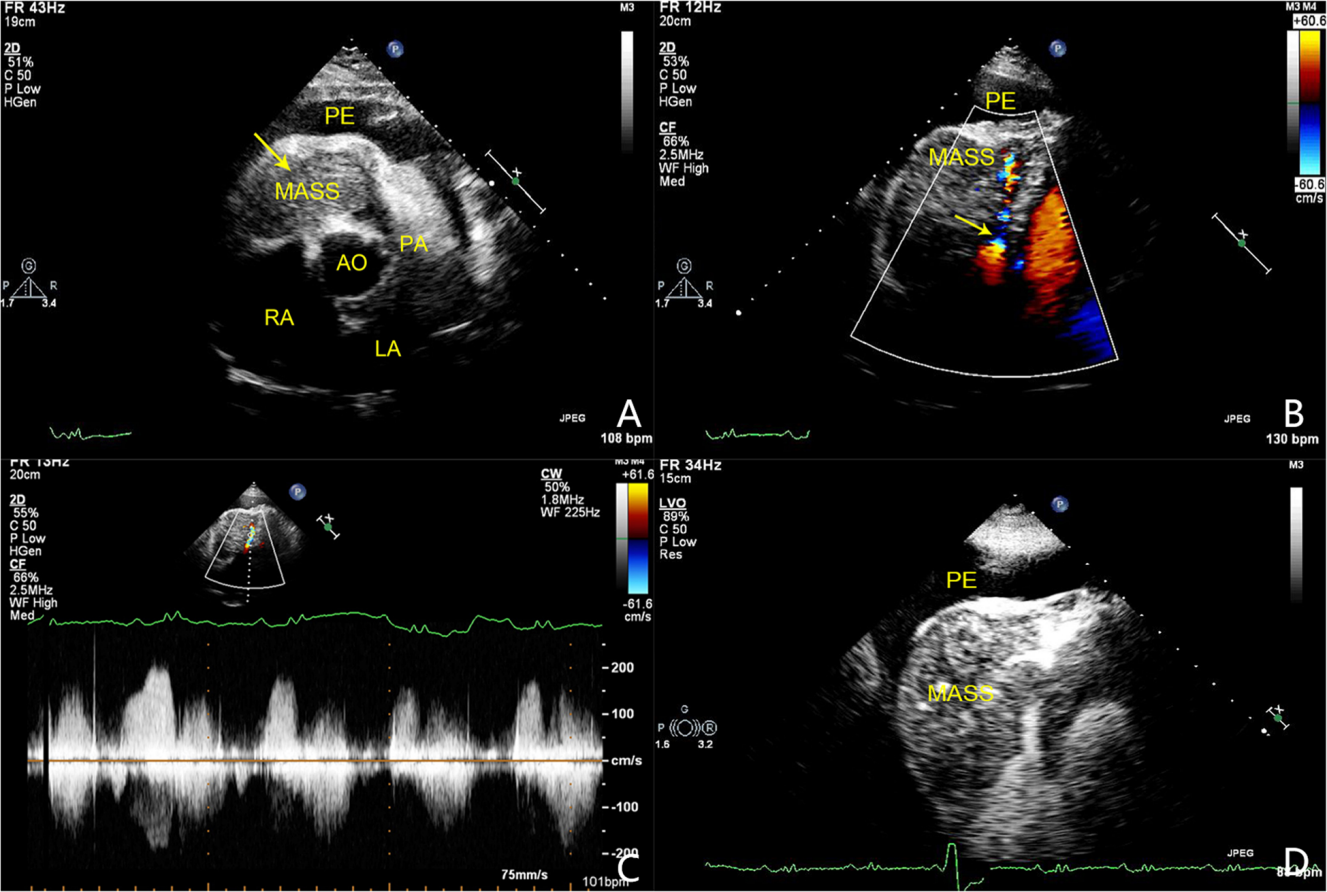
Echocardiographic findings. A huge mass occupying almost the whole RVOT (yellow arrow). Massive pericardial effusion can be observed. (A); Color Doppler showed fine and high velocity flow in cavity and inflow tract of RV. (B); Continuous-wave Doppler spectrum of the high-velocity flow in the inflow tract of RV, with the peak velocity of 2 m/s.(C); CE showed higher enhancement of contrast agent in the mass than the myocardium.(D). (AO: aortic artery, CE: contrast echocardiography, LA: left atrium, PA: pulmonary artery, PE:pericardial effusion, RA: right atrium)

**Fig. 2:**
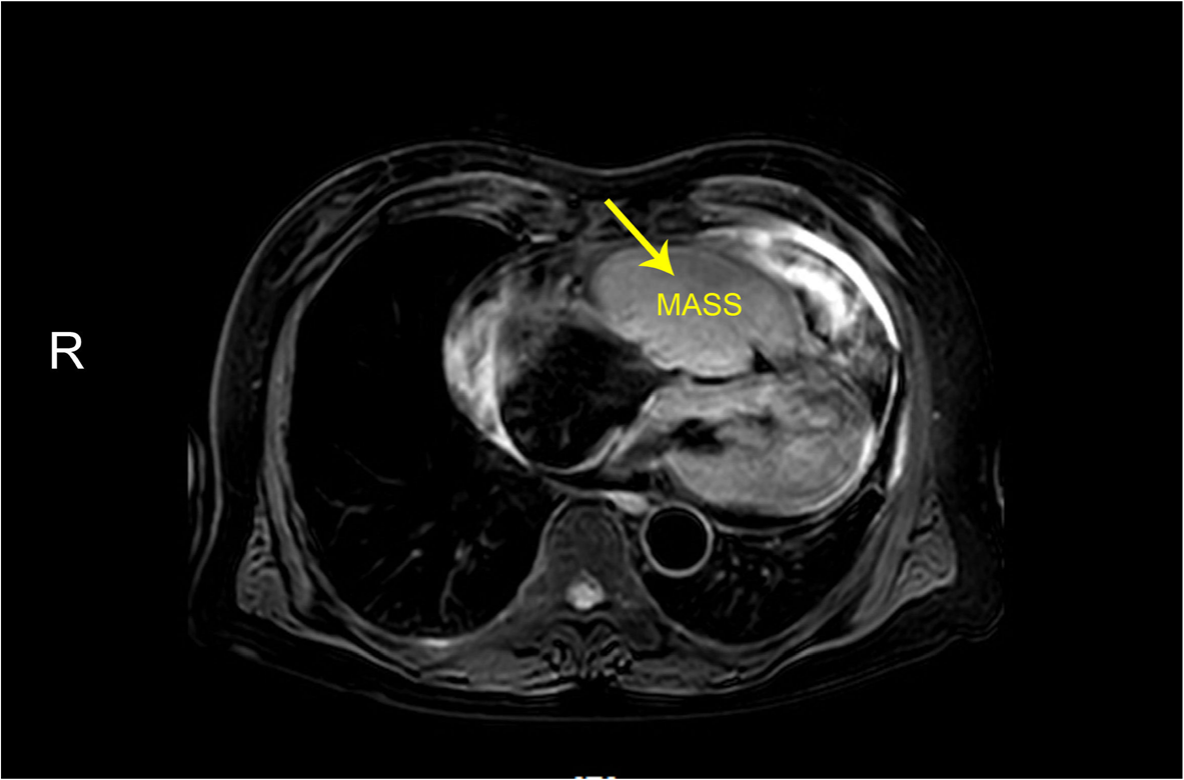
Cardiac MRI, T2 weighted image showed intracavity RV mass in lobular appearance, with blurred outline with myocardium

Positron emission tomography CT (PETCT) in other hospital showed that there was no nodule in the liver but some sporadic nodules in the lung. Large amount of pericardial effusion could also be observed. Transthoracic echocardiography (TTE) revealed a huge mass with moderate echogenicity in right ventricle (RV) which caused severe inflow obstruction. The mass blocked almost the whole cavity and showed little degree of mobility along with the cardiac cycle. The right ventricle outflow tract (RVOT) was also partially occupied, however, the speed of pulmonary artery (PA) remained normal (1.0 m/s). Contrast echocardiography (CE) demonstrated higher perfusion in the mass than in the myocardium after the administration of contrast agent. Cord-like vascular perfusion was observed in the mass. Color Doppler imaging showed fine and high velocity flow with the speed of 2 m/s in RV. Enlarged RV, RA and PA were also noted. Massive pericardial effusion was detected with enlarged IVC.(Fig. 1) Cardiac MRI confirmed that the huge mass had a size of 93x50x81mm and appeared lobular, and it seemed to have blurred the outline of anterior and lateral wall of RV.(Fig. 2) As his physical condition deteriorated rapidly, a cardiac surgery was operated to relieve the fatal obstruction. Fortunately, the patient was discharged one week after the surgery because of great improvement in his physical condition.

## Discussion and conclusion

The incidence of secondary tumor metastasis to heart is reported to be 1.5–21%. The metastasic tumors include melanoma which is of the highest incidence, renal cell carcinoma, breast, lung, thyroid and thymic carcinoma [2]. Other metastatic cardiac tumors are lymphomas and various sarcomas. Of patients with HCC, 5–10% will have cardiac metastasis. We reviewed a series of recent studies of isolated metastasis of HCC in RV between 1980 and 2018 and summarized relative outcomes.

In all 13 patients with the diagnosis of isolated RV metastasis HCC were identified. A summary of the patients is presented in Table 1. There were 5 females and 8 males with an age ranging from 43 to 79 years old. The conditions of RV obstruction caused by the mass include inflow (1), inflow and outflow (4), outflow (6) and 2 unknown. Very rarely, patient in our case only had inflow obstruction without outflow obstruction. The history of HCC ranged from 1 year to 10 years, however, it may have less correlation with metastasis to the cardiac chamber. Almost all the cases had myocardial infiltration. It testified the severe damage of myocardium caused by the mass, which may jeopardize cardiac function gradually in a rather silent way without specific symptoms. Treatments for the cardiac mass were mainly surgery and the survival time ranged from death after the surgery to 9 months. Therefore the prognosis remains rather poor and treatments are for relief of symptoms.

**Table 1:** Summary of patients presenting with HCC with isolated RV metastasis

Study	Sex	Age	Size (mm)	RV Obstruction	Myocardial infiltraion	Multimodality imaging	History of HCC	Treatment for HCC	Treatment for cardiac mass	Survival time
Steffens et al. (1980) [3]	M	60y	–	Inflow, outflow	(+)	TTE, angiography	6	Resection	surgery	Death after surgery
Lei et al. (1992) [4]	M	54y	–	–	(+)	–	–	–	surgery	7 months
Kotani et al.(2000) [5]	F	67y	43 × 37	Outflow	(+)	TTE, CT, angiography	3	Resection	transcoronary chemoembolization	Discharge
Longo et al.(2004) [6]	M	43y	–	–	(+)	X-ray, TTE,CT	3	Resection	–	1 month
Lin et al.(2004) [7]	M	45y	–	Outflow	–	–	–	chemotherapy	surgery	3 months
Chieng et al.(2005) [8]	F	65y	70 × 65	Outflow	(+)	US, CT, angiogram	1	Resection	surgery	3 months
Liu et al.(2006) [7]	F	45y	–	–	–	–	–	–	Surgery, chemotherapy	4 months
Kan et al.(2008) [7]	F	74y	–	Inflow, outflow	(+)	TTE, angiography	2	chemoembolization	surgery	4 months
Liu et al.(2010) [9]	F	46y	39.1 × 60.2	Inflow, outflow	(+)	TTE, CT	1.17	Resection, chemoembolization	surgery	4 months
Tameda et al.(2014) [10]	M	74y	–	Inflow, outflow	(+)	TTE, CT, MRI, PETCT	4	Resection, chemoembolization	No surgery	9 months
Lee et al.(2015) [2]	M	73y	–	Outflow	(+)	TTE, CT, angiography	4	Resection	surgery	–
Compagnoni et al.(2015)	M	54y	37 × 23	Outflow	(+)	TTE, CT, MRI	1	Liver transplant	Surgery, chemotherapy	–
Kim et al.(2016) [1]	M	79y	49.7 × 32.2	Outflow	(+)	TTE, CT, MRI, PETCT	10	Resection	surgery	–
Present study	M	63y	93x50x81	Inflow	(+)	TTE, CE, MRI, PETCT	2	Resection, chemoembolization	surgery	–

History of HCC: the time period patients suffered from HCC; −: not mentioned in the article; *TTE* Transthoracic echocardiography, *PETCT* Positron emission tomography CT, *US* other Ultrasonography

**Fig. 3:**
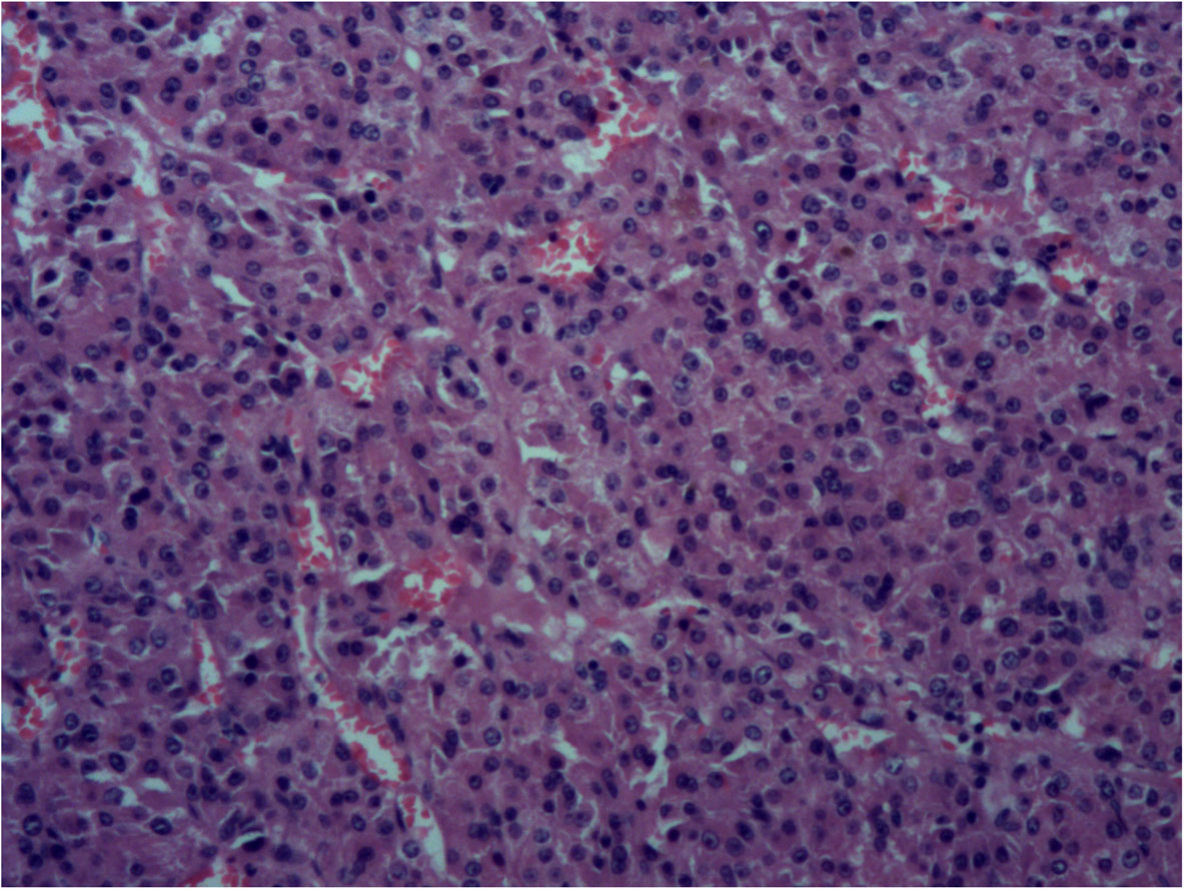
Histopathological findings of the mass showed tumor cells with markedly enlarged nucleus, pink-stained cytoplasm, gathering in cords or nests with intercellular sinusoids and necrosis (Hematoxylin and Eosin stain × 100)

In the diagnosis of metastasis HCC to heart, multimodality of cardiac imaging is of vital importance. The diagnosis might be neglected because of non-specific symptoms. According to previous cases, the patients lacked evident symptoms after a segmental hepatectomy or interventional chemotherapy and were found to have a large mass invaded in the cardiac cavities already. Yet, there may be no tumor recurrence in the liver. In our case, PETCT in other hospital didn’t provide information about the mass but only pericardial effusion. TTE, as the first means to find the mass, illustrated the contour, nature and mobility of the tumor. The high velocity flow of color Doppler also indicated inflow obstruction in RV. Moreover, higher enhancement of the mass and vascular perfusion in CE highly suggested malignancy. The huge mass in RV occupying nearly the whole cavity which caused inflow obstruction was confirmed by the surgery. The combination of TTE and CE displayed the mass in a relatively accurate and vivid view. Cardiac MRI provided more detailed information of the mass afterwards, which further confirmed and supported the diagnosis. Based on multimodality cardiac imaging findings, the patient underwent a successful operation of cardiac neoplasm. Surgical findings showed the tricuspid valves were severely blocked and the mass had partially infiltrated into the myocardium. The histopathological findings showed tumor cells growing in distorted version of normal architecture of various sizes. Tumor cells gathered in cords or nests with huge round nucleus, markedly pink-stained cytoplasm. Intercellular sinusoids and necrosis could also be clearly observed. The immunohistochemical findings proved that the mass was hepatocyte-derived and highly malignant.(Fig. 3).

HCC with RV metastasis without IVC and right atrium involvement is exceedingly rare and it may result from hematogenous spread. The prognosis of HCC with RV metastasis is generally poor. Surgical intervention may be palliative treatment to relieve lethal hemodynamic instability, however, the general consensus also believed that the effect of surgery was limited with untreated primary focus [11]. .In our case, chemotherapy was suggested after surgery, but unfortunately the patient refused any further treatment. Besides, transcoronary chemoembolization has also been regarded as a valuable choice. At present, standard treatment has not been well established for metastatic HCC with invasion into the RV [10]. To improve a poor prognosis of metastatic HCC, various approaches combining chemotherapy, radiotherapy, and surgery are needed for both primary and metastatic lesions. These palliative treatments may probably alleviate life-threatening symptoms and prolong survival time to some degree.

## Data Availability

All data that was generated or analyzed during the current study are available from the corresponding author on reasonable request.

## Abbreviations

BilD: Direct bilirubin
BilT: Total bilirubin
CE: Contrast echocardiography
CKMB: Creatine kinase isoenzymes
CRP: C reaction protein
CT: Computed tomography
GGT: Glutamyl transpeptadase
HCC: Hepatocellular carcinoma
IVC: Inferior vena cava
MRI: Magnetic resonance imaging
PA: Pulmonary artery
PETCT: Positron emission tomography CT
RA: Right atrium
RV: Right ventricle
RVOT: Right ventricle outflow tract
TnI: Troponin I
TTE: Transthoracic echocardiography
US: Other ultrasonography
